# Frailty and pituitary surgery: a systematic review

**DOI:** 10.1007/s11102-025-01507-2

**Published:** 2025-03-17

**Authors:** Mendel Castle-Kirszbaum, Ann McCormack, Christopher Ovenden, Jeremy Kam, James King, Yi Yuen Wang, Tony Goldschlager

**Affiliations:** 1 Department of Neurosurgery, Monash Health, 246 Clayton Road, Clayton VIC 3168, Melbourne, Australia; 2 Department of Surgery, Monash University, Melbourne, Australia; 3 Faculty of Medicine, University of New South Wales, Sydney, Australia; 4 Department of Endocrinology, St Vincent’S Hospital, Sydney, Australia; 5 Department of Neurosurgery, Royal Adelaide Hospital, Adelaide, Australia; 6 Faculty of Health and Medical Sciences, Adelaide Medical School, Adelaide, Australia; 7 Department of Neurosurgery, Royal Melbourne Hospital, Melbourne, Australia; 8 Department of Surgery, University of Melbourne, Melbourne, Australia; 9 Department of Neurosurgery, St Vincent’S Health, Melbourne, Australia

**Keywords:** Frailty, Pituitary, Surgery, Transsphenoidal, Cushing’s disease, Acromegaly, Length of stay, Complication

## Abstract

**Background:**

Frailty is a state of physiological vulnerability rendering patients susceptible to adverse perioperative outcomes after neurosurgery. The effect of frailty on surgical success and complication rates in patients undergoing transsphenoidal pituitary surgery is unclear.

**Methods:**

A systematic review of the literature was performed in accordance with the PRISMA statement. Studies that utilised validated metrics to report the effect of frailty on pituitary surgery were included.

**Results:**

A total of 13 studies were included, comprising 124,989 patients. Frailty was exclusively assessed with cumulative deficit metrics, however there was significant heterogeneity in patient population, frailty definitions and assessment, and outcomes. Frail patients undergoing transsphenoidal surgery experienced higher rates of medical complications, resulting in longer hospital stays, greater hospitalisation costs, higher rates of unplanned readmission, more discharges to a destination other than home, and increased mortality. These outcomes directly correlated with increasing degrees of frailty. Surgical outcomes were not affected by frailty, with similar rates of biochemical remission, visual recovery, and improvement in quality of life.

**Conclusion:**

Frailty is seen in a minority of patients undergoing pituitary surgery, but is an important indicator of perioperative risk. Frailty assessment should not be used as a reason to withhold surgery, but rather to predict and mitigate perioperative complications to improve outcomes in pituitary surgery.

**Supplementary Information:**

The online version contains supplementary material available at 10.1007/s11102-025-01507-2.

## Introduction

Frailty is a state of physiological vulnerability rendering patients susceptible to adverse perioperative outcomes. Distinct from aging, this reduced tolerance to stress has consistently correlated with perioperative morbidity in large neurosurgical cohorts[1, 2]. Whether frailty has the same effect on postoperative outcomes in patients undergoing transsphenoidal surgery is unclear. The transsphenoidal approach is the preferred surgical corridor for sellar and many parasellar tumours, and has a favourable morbidity profile[3, 4]. Here, we review the literature on frailty assessment in pituitary surgery, establishing its role in prospective identification of at-risk patients to guide patient selection, surgical decision making, and postoperative care.

## A primer on frailty definitions

Frailty is a syndrome characterised by diminished endurance, strength, homeostatic capabilities, and physiological reserve that increases vulnerability for developing dependency and adverse health outcomes[5]. The conceptualisation and operationalisation of frailty is to define physiological vulnerability that is not captured by disease diagnoses or chronological age[6]. Clinically, the frailty phenotype is defined by unintentional weight loss, sarcopenia, weakness, slowness, exhaustion, poor endurance, and physical inactivity[7]. Although this phenotypic definition is identifiable in an individual patient, the need for objective, reproducible assessment in large cohorts led to the parallel characterisation of frailty as an accumulation of medical comorbidities, the cumulative deficit model or frailty index[8–10]. This approach defines frailty as a stochastic accumulation of structural and functional defects in a range of physiological systems, and operationalises it as a simple tally of these deficits[11]. Cumulative deficit models predominate in the neurosurgical literature[12], presumably because their components are readily extractable from electronic records and thus can be implemented widely and retrospectively. The literature lacks consensus as to the optimal definitions, metrics, and cutoffs to characterise frailty. Regardless of the definition used, frail patients experience worse perioperative outcomes and greater mortality[13–15].

## Methods

A systematic search of the literature was conducted using the Medline and PubMed databases in accordance with the Preferred Reporting Items for Systematic reviews and Meta-Analyses (PRISMA) statement[16]. All studies from database inception until November 2024 were queried using the search string:

(Frail* OR frailty/ OR geriatric*) AND (Pituitary Diseases/ OR Pituitary ACTH Hypersecretion/ OR ACTH-Secreting Pituitary Adenoma/ OR Pituitary Apoplexy/ OR Growth Hormone-Secreting Pituitary Adenoma/ OR Pituitary OR Pituitary Neoplasms/ OR acromegaly/ OR Cushing* disease OR transsphenoidal OR endonasal).

Exclusion criteria were single case reports, studies published in languages other than English, and studies where data specific to pituitary pathology could not be isolated. The references of included studies were also hand-searched to identify additional eligible studies.

After title and abstract review, full-text review was performed in appropriate studies to determine suitability for inclusion by a reviewer (MCK) and confirmed by a second reviewer. Inclusion criteria were defined as: 1. Trials, prospective and retrospective cohort studies that report the outcomes of patients undergoing surgery for pathology related to the pituitary gland and sellar region; 2. Use of a validated frailty metric to identify or grade frailty within the patient population. 3. Comparison of outcomes between frail and non-frail groups.

Included studies underwent independent data extraction including study year, study size, surgical technique, frailty metric, definition of frailty, and outcomes.

## Results

In total, 13 studies were identified from the systematic literature search (Supplementary Fig. 1), comprising 124,989 patients (Tables 1 and 2). The literature consisted mostly of retrospective studies (11/13, 85%). The majority of these (8/11, 73%) were analyses of large American multi-institution perioperative databases: The National Surgical Quality Improvement Program (NSQIP)[17–21], National Readmission Database (NRD)[22], and the National (Nationwide) Inpatient Sample (NIS)[23, 24]. Prospective studies were infrequent (2/13, 15%) and represented a relatively small proportion of the overall literature (*n* = 364). Pathology included pituitary adenomas (neuroendocrine tumours) and parasellar tumours including craniopharyngiomas and meningiomas but were often not specifically reported. There was significant heterogeneity in patient population, method of assessing frailty, cutoffs for defining frailty, and outcome assessment. Overall, studies were at high risk of bias.

**Table 1:** – Characteristics of studies assessing frailty in patients undergoing pituitary surgery

Author (Year)	Study Type	N	Age (Frail vs Not)	Pathology	Surgical Technique	Frailty Metric
Asemota (2020)	Retro database	115,317	57 ± 17y vs 52 ± 16 (p < 0.01)	NR	TS (NOS)	ACG
Castle-Kirszbaum (2021)	Pros cohort	304	58 ± 15y vs 53 ± 17y (p = 0.05)	PA (mixed functioning and NFPA)	ETS	mFI-5, mFI-11, CCI Frail—mFI-5 ≥ 2
Findlay (2024)	Retro cohort	318	49 ± 12y vs 40 ± 14y (p < 0.01)	CD	ETS and MTS	mFI-5, mFI-11, RAI-A Frail—mFI-11 ≥ 4; Managing well—mFI-11 = 2–3
Khalafallah (2020)	Retro cohort	234	NR	PA (mixed functioning and NFPA)	ETS	mFI-5 No cutoff
Kshirsager (2024)	Retro cohort	88	NR	Suprasellar (meningioma, craniopharyngioma, PA, RCC)	ETS	mFI-5, CCI Frail—mFI-5 ≥ 1
Marquez (2023)	Pros cohort (LTTE)	60	NR	PA (NOS)	ETS	RAI-C
Martin (2022)	Retro database	1926	60 ± 12 vs 47 ± 15 (p < 0.01)	NR	ETS and MTS	mFI-5 Groups mFI = 0, 1, 2–5
Nguyen (2021)	Retro database	993	60 ± 13 vs 53 ± 15 (p < 0.01)	NR	MTS	mFI-11 Frail – mFI-11 ≥ 2
Peterson (2022)	Retro database	1415	43 ± 22 vs 38 ± 22 (p = 0.01)	Craniopharyngioma	NR	HFRS Low risk = 0–5, intermediate risk 6–15 High risk > 15
Shahrestani (2021)	Reto database	746	63.7 vs 62.5 (aged matched)	PA (mixed functioning and NFPA)	ETS and MTS	ACG
Sukys (2022)	Retro database	680	NR	NR	ETS	mFI-11, CCI Groups mFI-11 = 0, 1, 2, ≥ 3
Thommen (2022)	Retro database	1454	NR	PA (NOS)	TS and TC	mFI-5 Groups mFI = 0, 1, 2, ≥ 3
Varela (2024)	Retro database	1454	NR	PA (NOS)	TS and TC	mFI-5, RAI-A Groups mFI = 0, 1, 2, ≥ 3 Groups RAI-A = ≤ 10, 11–20, 21–30, ≥ 31

*TC* Transcranial, *TS(NOS)* Transsphenoidal not otherwise specified, *ETS* Endoscopic transsphenoidal, *MTS*  Microscopic transsphenoidal, *Retro* Retrospective, *pros* Prospective, *PA* Pituitary adenoma, *RCC* Rathke Cleft cyst, *LTTE* Letter to the editor, *HFRS* Hospital Frailty Risk Score , Johns Hopkins *ACG* Adjusted Clinical Groups

**Table 2:** – Outcomes of studies assessing frailty in patients undergoing pituitary surgery

Author (Year)	Unplanned Readmission	LOS	Mortality	Medical Complication	Surgical complication	Non-home DC	Comment
Asemota (2020)	NR	9.3 ± 14d vs 4.5 ± 5d (p < 0.01)	1.46% vs 0.37% (p < 0.01)	AMI—1.8% vs 0.3% (p < 0.01) DVT/PE—1.4% vs 0.5% (p < 0.01) AKI—5.4% vs 1.0% (p < 0.01)	Water Balance Disorder – 16.2% vs 13.5% (p = 0.15) Stroke – 5.0% vs 1.3% (p < 0.01) CSF leak – 9.3% vs 8.3% (p = 0.50)	61.6% vs 37.9% (p < 0.01)	Frail patients with greater comorbidities (CCI) had more compications
Castle-Kirszbaum (2021)	NR	NR	2.4% vs 0% (NS)	AMI – 2.4% vs 0.8% (NS) DVT/PE – 0.0% vs 0.4% (NS)	Visual worsening – 0.0% vs 1.3% (NS) New hypopituitarism – 46.2% vs 22.0% (p = 0.06)	NR	Frailty had no effect of postoperative QoL
Findlay (2024) *	16.7% vs 8.2% (p = 0.27)	5.3 ± 3d vs 3.7 ± 2d (p = 0.02)	0.0% vs 0.07% (NS)	AMI – 2.4% vs 0.8% (NS) DVT/PE – 8.3% vs 1.3% (NS)	GTR = 83% vs 83% (NS)	25% vs 1.9% (p < 0.01)	Frailty had no association with remission (68.6% vs 72.9%, p = 0.45) Functional status (within the mFI) was the strongest predictor of LOS and disposition Frailty was not associated with remission rates (68.6% vs 72.9%, p = 0.45, authors calculations)
Khalafallah (2020)	NR	NR	NR	NR	NR	NR	Increasing mFI-5 score correlated with increasing LOS (p < 0.01), and healthcare costs (p < 0.01) but not readmission (p = 0.09)
Kshirsager (2024)	OR 2.35 (95%CI: 1.10–5.64. p = 0.04)	NS (p = 0.30)	NR	NS (p = 0.30)	NS (p = 0.14)	NR	Age, CCI, mfI-5, and ASA all did not correlate with complications
Marquez (2023)	NR	NR	NR	NR	NR	NR	Rate of adverse events correlated with increasing RAI-C Score: score 0–10 with 7.7%, 11–20 with 11.1%, and 21 + with 25%
Martin **(2022)	7.7% vs 7.9% (NS)	5.8 ± 11 vs 4.2 ± 7 (p < 0.01)	1.0% vs 0.5% (NS)	7.7% vs 4.4% (p = 0.02)	4.7% vs 2.3% (p = 0.03)	9.9% vs 2.3% (p < 0.01)	
Nguyen (2021)	NR	5.8 ± 7 vs 4.5 ± 7 (p = 0.02)	3.0% vs 0.6% (p = 0.02	8.3% vs 4.8% (NS)	1.2% vs 1.1% (NS)	NR	
Peterson (2022) ***	NR	18.8 ± 20 vs 7.3 ± 5 (p < 0.01)	3.8% vs 0.0% (p < 0.01)	Any complication – 78.5% vs 63.2% (p < 0.01)	NR	58.2% vs 16.7% (p < 0.01)	
Shahrestani (2021)	30d = 8.9% vs 7.9% (p = 0.5) 90d = 17.8% vs 10.7% (p < 0.01)	13.8 ± 19 vs 4.4 ± 5 (p < 0.01)	NS	NS	NS	NS	Frail patients had higher inpatient hospital costs (p < 0.01). No difference in rates of CSF leak, dural tear, SIADH, and anaemia
Sukys (2022)**	6.4% vs 4.0% (NS)	NR	2.1% vs 0.7% (NS)	NS	NS	NR	ASA score had stronger correlation to ICU-level complications than mFI
Thommen (2022)	NR	NR	NR	NR	NR	NR	Increasing mFI was associated rate of with major complication, unplanned readmission, hospital LOS, and discharge to a nonhome destination, but not mortality
Varela (2024)	NR	NR	NR	NR	NR	NR	RAI was superior to the mFI-5 at predicting extended LOS

^*^ = frail vs combined fit and managing well groups, authors calculations

^**^ = mFI = 0 and mFI = 1 vs mFI 2–5, authors calculations

^***^ = Low risk vs intermediate + high frailty risk, authors calculations

Frailty was measured with several metrics across included studies (Supplementary Data 2):

- Metrics based on accumulated comorbidities: 5 Factor Modified Frailty Index (mFI-5) [8], 11 Factor Modified Frailty Index (mFI-11) [9], Hospital Frailty Risk Score (HFRS) [10], Charlson Comorbidity Index (CCI) [25]
- Metrics based on the presence of specific “frailty defining” disease states: Johns Hopkins Adjusted Clinical Groups frailty defining illnesses (ACG) [26]
- Metrics based on a combination of functional status and accumulated comorbidities: Risk Analysis Index – Clinical (prospective) (RAI-C) [27], Risk Analysis Index – Administrative (retrospective) (RAI-A) [27].

Cumulative deficit models were used in all studies. The Modified Frailty Index (mFI) was the most common cumulative deficit metric (69%, 9/13) (mFI-5 = 46%, 6/13; mFI-11 = 31%, 4/13), followed by the CCI (23%, 3/13), and HFRS (8%, 1/13). The ACG was used in a single study (8%, 1/13) and defines frailty by the presence of one of more “frailty defining” illnesses, while the RAI combines accumulated comorbidities and phenotypic frailty assessment, including nutrition, mobility and functional status, and was used in three studies (23%, 3/13).

Frailty was variably graded across studies, including binary (presence or absence) (5/13, 38%), ordinal (6/13, 47%), and continuous (2/13, 15%) definitions. Cutoffs for binary and ordinal groups using the same metrics differed between studies[18, 28, 29]. In large pituitary surgery database studies, 1–2% of patients were considered frail[23]. Frailty scores were higher in Cushing’s disease and acromegaly compared to prolactinoma and non-functioning adenomas[30], with frailty seen in up to 42% of Cushing’s disease and 16% of acromegaly, compared to 8% of non-functioning tumours[28]. There was good correlation between different frailty metrics when used in the same cohort[31].

Hospital length of stay (LOS) was longer in frail patients[17, 18, 22–24, 31], with greater degrees of frailty correlating to longer length of stay[20, 30]. In the largest study, LOS was twice as long for frail patients (9.3 ± 14 vs 4.5 ± 5 days, p < 0.001) (Mean ± SD)[23].. Preoperative functional status was the strongest predictive component of admission duration within frailty metrics[31]. The RAI-A was superior to the mFI in predicting extended LOS, although the absolute discriminative capacity was moderate (C-statistic = 0.59)[21]. In patients with functional tumours, particularly Cushing’s disease, the mFI demonstrated good discriminative capacity for LOS, possibly due to the inclusion of diabetes mellitus as a criterion for frailty [30, 31]. Microscopic transsphenoidal techniques were less common in frail patients with Cushing’s disease, however the association of frailty with LOS was not effected by surgical technique[31].

The association of frailty with extended hospital LOS appeared independent of patient age, Knosp grade, surgeon experience, surgical technique, or American Society of Anesthesiologists (ASA) grade [17, 20, 31]. LOS was also associated with functional tumours, Cushing’s disease[30], malnutrition[22], complications[31], and HbA1c level[31]. Hospital costs were also higher in frail patients[22, 30].

Unplanned readmission was not consistently associated with frailty[17, 19, 29, 31], however there was a trend for increasing rates of readmission in the most frail patients[20, 30], and at later time points[22]. The association of frailty with unplanned readmission appeared independent of patient age and ASA grade[29, 31].

Inpatient mortality was low overall, 0.39% in the largest study[23]. Frailty was associated with greater mortality rate in the majority of studies[18, 19, 23, 24, 28], and was independent of age and body mass index (BMI)[18].

Frailty was consistently associated with discharge to a non-home facility[17, 20, 23, 24, 31]. Increasing grade of frailty was associated with a higher rate of non-home discharge[20, 24], independent of age, Knosp grade, ASA grade, surgical technique, and surgeon experience [20, 23, 31]. Preoperative functional status, and a history of myocardial infarction, chronic obstructive pulmonary disease (COPD), and diabetes mellitus were the strongest component predictors of discharge to a non-home facility within frailty metrics[31].

Medical complications were generally more frequent in frail patients[17, 18, 20, 23, 24, 31, 32]. Perioperative respiratory, renal, neurovascular, and cardiac complications were associated with increasing degrees of frailty[17, 23, 24], independent of age[20, 23, 24].

ASA grade was a stronger predictor of medical complications than frailty metrics[17–19].

Surgical complications were not consistently associated with frailty[17, 29]. Arginine vasopressin deficiency (AVP-D, diabetes insipidus) and other water balance disorders[22, 23, 28, 29, 31, 31, 33], new hypopituitarism [23, 28, 29], and postoperative cerebrospinal fluid (CSF) leak [22, 23, 31, 33] were all similar between frail and non-frail cohorts.

Surgical outcomes were infrequently reported. Frailty was not associated with biochemical remission rates in patients with Cushing’s disease[31] and other functional tumours[28]. Similarly, frailty had no effect on visual field recovery[28, 29]. Overall and sinonasal-specific quality of life improved after endoscopic pituitary adenoma surgery, and was not affected by frailty[28].

## Discussion

Frail patients undergoing transsphenoidal surgery experience higher rates of medical complications, resulting in longer hospital stays, greater hospitalisation costs, higher rates of unplanned readmission, and more discharges to a destination other than home. These outcomes directly correlated with increasing degrees of frailty. Despite an overall low rate, perioperative mortality was substantially higher in frail patients. Surgical outcomes were not affected by frailty, with similar rates of biochemical remission, visual recovery, and improvement in quality of life.

Standardised definitions to identify and grade frailty are lacking. Characterised by impaired resilience to physiological stress, the frailty syndrome is recognised by weakness, weight loss, poor endurance, slowness, and physical inactivity[7]. The existence of the frailty syndrome implies an underlying pathophysiology responsible for the development and progression of the phenotype. These mechanisms include malnutrition, neuroendocrine dysregulation, chronic inflammation, immunodeficiency, and cell senescence. There is likely significant overlap with the biology of aging[34]. Cumulative deficit metrics to define frailty predominate the literature due to simplicity, ease of retrospective implementation, and objectiveness[8–10]. These competing cumulative deficit and phenotype frameworks identify different subsets of frail patients, suggesting not all frailty is equal. However, both cumulative deficit and phenotype models appear adequate surrogates for physiological reserve, particularly in the frailest patients [35, 36]. In this review, frailty was exclusively defined by comorbidity burden. These metrics were developed retrospectively from large databases of predominantly non-neurosurgical hospitalisations (e.g. NSQIP for the mFI), and metric components are inherently limited to the datapoints collected in each database. Despite this, frailty defined by cumulative deficits models has consistently correlated with poor outcomes after neurosurgery[1, 2].

Advanced age is frequently used as a surrogate for frailty, yet they are not synonymous. Although age has been associated with poorer outcomes in pituitary adenoma surgery[37], appropriately selected patients even in their ninth decade of life can achieve excellent outcomes[38, 39]. Frailty is a more specific predictor of perioperative risk [17, 18, 20, 23, 40]. Distinguishing frailty from chronological age is important, as it identifies modifiable risk factors that can be optimised[22]. Functional status and ASA grade also predict medical complications[17]. The ASA grade measures current physiological status and may be more reliable predictor of acute medical complications[33]. Frailty metrics that include and emphasise functional status appear the best predictors of length of stay, mortality, and discharge destination [21]. Addition of biochemical markers, including haemoglobin[41] and albumin[42, 43], as well assessment of nutritional status[44] and sarcopenia[45, 46], could be used to augment current models.

Frailty is of particular relevance to pituitary surgery given many components of the frailty syndrome may be aetiologically linked to endocrinopathy[47]. Sarcopenia and poor bone health is associated with growth hormone (GH), cortisol, and androgen deficiency[48] and excess[49, 50], while reduced vitality can be associated glucocorticoid, androgen, and thyroid hormone deficiency[51]. Frailty has been associated with deficiency and impaired rhythmicity of glucocorticoid secretion[52–54], androgen deficiency, GH/insulin-like growth factor 1 (IGF-1) deficiency[55, 56], diabetes mellitus, and the metabolic syndrome [48, 55]. Frailty is also common in patients with functioning tumours[28, 31], particularly Cushing’s disease and Acromegaly. Comorbidities included in cumulative deficit models are frequently seen in functioning pituitary tumours, such as hypertension, diabetes mellitus, cardiovascular and cerebrovascular disease, where they tend to be severe and treatment resistant[57]. These comorbidities do not resolve instantaneously, and may persistent many years after biochemical remission[58–60]. Patients with Cushing’s disease are at high risk of postoperative water balance disorders [61] and hypocortisolism[62], which may increase length of stay and readmission rates. Perioperatively, they represent some of the most physiologically fragile patients in neurosurgery due to impaired wound healing[63], susceptibility to infection[64], myopathy[60], cardiovascular[65] and venous thromboembolic risk[66], and increased all-cause mortality[67]. The association of frail patients harbouring smaller pituitary adenomas is likely an epiphenomenon of the greater proportion of Cushing’s disease in frail cohorts. Patients with acromegaly harbour similar perioperative risks due to upper airway obstruction[68], diabetes mellitus, hypertension[69], cardiomyopathy and cardiovascular risk[70, 71], and increased all-cause mortality[72].

Transsphenoidal surgery improves quality of life for patients with pituitary adenomas[73–76] and other anterior skull base tumours[77–79]. Despite increased perioperative risk, frail patients experience equivalent biochemical, visual and quality of life outcomes from transsphenoidal surgery[28, 29, 31]. Thus, frailty assessment should not be used as a reason to withhold surgery, but rather to predict and mitigate perioperative complications to improve outcomes in pituitary surgery.

Frailty appears best assessed with comprehensive metrics that focus on functional status, as these demonstrate the strongest correlations with perioperative outcomes[21, 80, 81]. How frailty assessment can be incorporated into routine practice remains to be established. Identification of physiological vulnerability is important to neurosurgical decision making, and facilitates informed preoperative risk assessment, optimisation of modifiable comorbidities, targeted initiation of “prehabilitation”, and more effective utilisation of healthcare resources. Frail patients, when identified early in their journey, could be optimised preoperatively with intensive medical therapy, nutritional supplementation and physiotherapy involvement[82, 83]. After surgery, closer surveillance and physician involvement may mitigate perioperative complications, and planned discharge to non-home facilities may obviate logistical barriers that prolong length of stay beyond that required for medical stabilisation. Future prospective studies are required to establish how frailty assessment and preoperative optimisation can be incorporated into pituitary surgery and elucidate its effect on clinical outcomes.

The included studies have several limitations. Cumulative deficit metrics may have limited generalisability to pituitary surgery. Endocrinopathy is a principal driver of frailty in pituitary disease, and current metrics may not be sensitive to the physiological effects of hypopituitarism. The severity, chronicity, and disease control of comorbidities are also not considered. Patient characteristics, including tumour type, size, location, cavernous sinus invasion, previous treatment, and other factors that influence surgical time, case complexity, risk, and outcome, are not provided in all studies, particularly large database analyses. The definition, measurement, and threshold to characterise frailty is not consistent in the literature. Many frailty metrics are dichotomous, oversimplifying the spectrum of frailty. The ACG defines frailty by the presence of a single “frailty-defining diagnosis” such as severe visual impairment, likely overestimating frailty in the pituitary patient population. Temporal relations are also clouded in retrospective studies. Frailty defining comorbidities may be the result of surgery rather than contributing to complications; this distinction may be lost in large database studies. Database studies are also limited by the outcomes they record. The NSQIP, NIS, and NRD do not report neurosurgical specific outcomes, and postoperative outcomes and complications are only captured for the first 30 days. Surgical techniques were not compared, although the differences between endoscopic and microscopic approaches are unlikely to be significant[84]. No comparison with transcranial surgery was available. Surgical outcomes, such as extent of resection, were inadequately reported. The safety profile of other common neurosurgical procedures in frail patients was not explored. Finally, comparisons between studies were limited due to the use of different frailty metrics and thresholds for defining frailty. This also precluded data pooling and meta-analysis. These limitations serve as opportunities for improvement in future studies.

## Conclusion

Frailty is seen in a minority of patients undergoing pituitary surgery, but is an important marker of perioperative risk, particularly in functioning tumours. Frailty is associated with higher rates of medical complications, unplanned readmission, prolonged admission, discharge to non-home destination, and mortality. Surgical outcomes are equivalent, and frail patients experience similar improvements in quality of life to non-frail. Frailty assessment should not be used as a reason to withhold surgery, but rather to predict and mitigate perioperative complications to improve outcomes in pituitary surgery.

## Supplementary Information

Below is the link to the electronic supplementary material.

Supplementary file1 (TIFF 9670 KB)

Supplementary file2 (DOCX 2353 KB)

## Data Availability

Not applicable

## Data availability

No datasets were generated or analysed during the current study.
