# Supplementary figures and images for: Frailty and pituitary surgery: a systematic review
Source: Pituitary. 2025 Mar 17;28(2):43. doi: 10.1007/s11102-025-01507-2 (PMC11913960; doi:10.1007/s11102-025-01507-2)

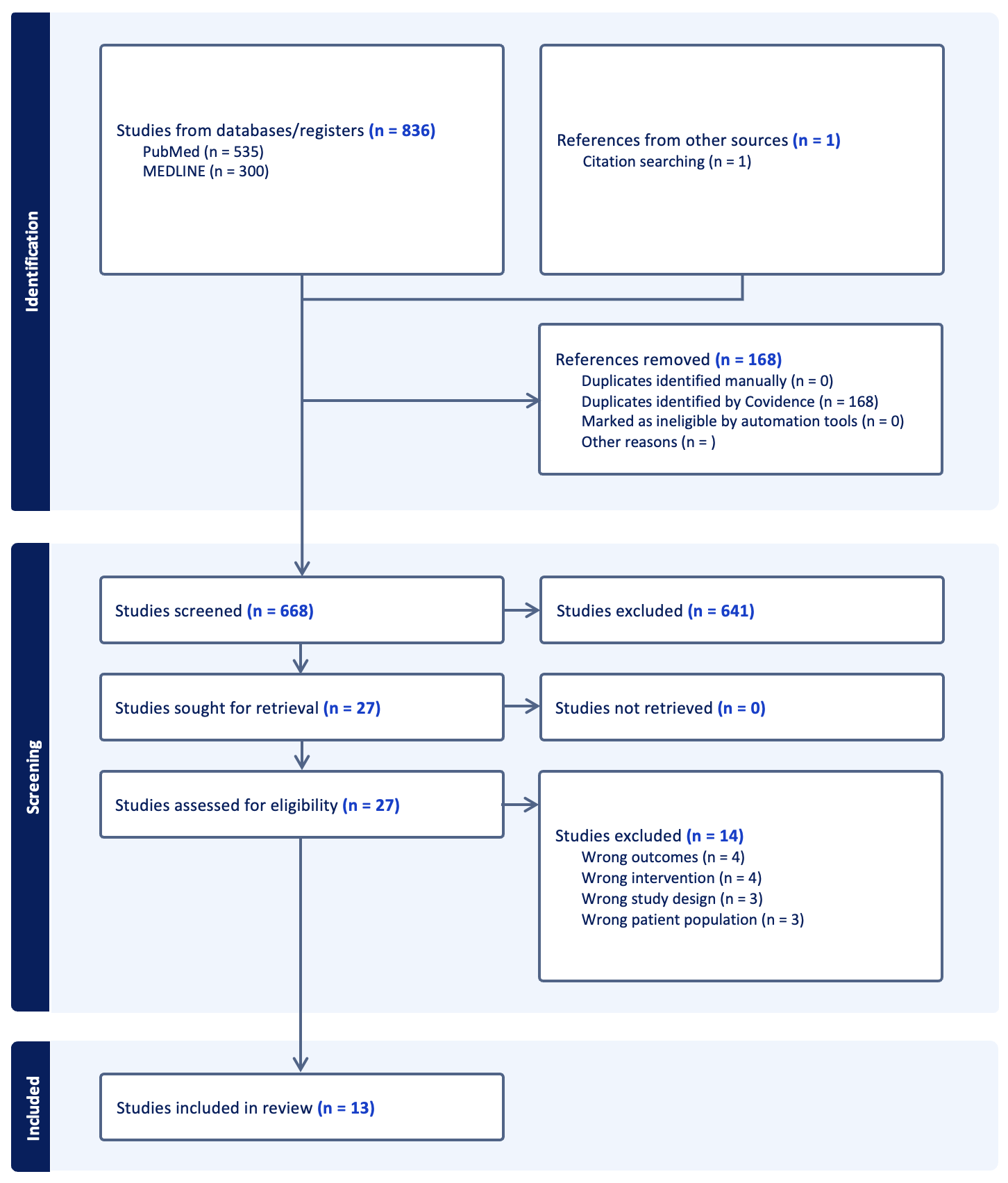

Supplement: Supplementary file 1 — Supplementary file1 (TIFF 9670 KB) [file 11102_2025_1507_MOESM1_ESM.tiff]
