# Supplementary material for: Frailty and pituitary surgery: a systematic review
Source: Pituitary. 2025 Mar 17;28(2):43. doi: 10.1007/s11102-025-01507-2 (PMC11913960; doi:10.1007/s11102-025-01507-2)
Supplement: Supplementary file 2 — Supplementary file2 (DOCX 2353 KB) [file 11102_2025_1507_MOESM2_ESM.docx]

**Supplementary Data 2 – Summary of Frailty Metrics**

The frailty metrics described in this review are

- 11 Factor Modified Frailty Index (mFI-11) [1]
- 5 Factor Modified Frailty Index (mFI-5) [2]
- Hospital Frailty Risk Score (HFRS) [3]
- Charlson Comorbidity Index (CCI) [4]
- Johns Hopkins Adjusted Clinical Groups frailty defining illnesses (ACG) [5]
- Risk Analysis Index (prospective) (RAI-C) [6]
- Risk Analysis Index (retrospective) (RAI-A) [6]

The are summarised below.

**11 Factor Modified Frailty Index (mFI-11)**

The 11-factor modified Frailty Index (mFI-11) was developed from a 70-item scale, the Canada Study of Health and Aging Frailty Index (CSHA-FI), which analysed nutrition, physical activity, cognition and comorbidities. Only 11 of the 70 items in the CSHA-FI were originally collected in the American College of Surgeons National Surgical Quality Improvement Program (NSQIP) database of 30-day morbidity and mortality. These 11 items became the mFI-11, with 1 point awarded for each comorbidity. There is no accepted cutoff between frail and non-frail using the mFI-11, and it can be used as a continuous variable, or binary with an arbitrary cut-off.

| **mFI-11** |
| --- |
| History of diabetes mellitus |
| Not independent in functional status |
| History of chronic obstructive pulmonary disease or pneumonia |
| History of congestive heart failure |
| Myocardial infarction |
| History of percutaneous coronary intervention, cardiac surgery, or angina |
| Hypertensive medication |
| Peripheral vascular disease or rest pain |
| Impaired sensorium |
| Transient ischemic attack or cerebrovascular accident |
| Cerebrovascular accident with deficit |

**5 Factor Modified Frailty Index (mFI-5)**

In 2015, the NSQIP database altered the datapoints that it collected, such that only five of the original 70 items from the CSHA-FI (and 5 of the 11 items of the mFI-11) remained. These remaining 5 items formed the mFI-5, with one point awarded for each comorbidity. Again, there is no accepted cutoff between frail and non-frail using the mFI-11, although most commonly either a score of ≥1 or ≥2 is used to define frailty.

| **mFI-5** |
| --- |
| History of diabetes mellitus |
| Not independent in functional status |
| History of chronic obstructive pulmonary disease or pneumonia |
| History of congestive heart failure |
| Hypertension |

**Hospital Frailty Risk Score (HFRS)**

The hospital frailty risk score was developed by identifying a group of patients >75 years old who had high hospital resource use and length of stay, and had diagnoses that were a priori considered to be associated with frailty. The comorbidities of this group were then analysed. The HRFS uses a weighted score of 109 diagnoses (ICD-10 codes) to produce a score. The HFRS was classified into low-risk (HFRS <5), intermediate risk (HFRS 5-15), and high risk (HFRS >15).

| Condition | Risk Score | Condition (Cont) | Risk Score (Cont) | Condition (Cont) | Risk Score (Cont) |
| --- | --- | --- | --- | --- | --- |
| Dementia in Alzheimer's disease | 7.1 | Other functional intestinal disorders | 1·8 | Diarrhoea and gastroenteritis of presumed infectious origin | 1·1 |
| Hemiplegia | 4.4 | Acute renal failure | 1·8 | Pneumonia, organism unspecified | 1·1 |
| Alzheimer's disease | 4.0 | Decubitus ulcer | 1·7 | Pneumonitis due to solids and liquids | 1·0 |
| Sequelae of cerebrovascular disease | 3.7 | Carrier of infectious disease | 1·7 | Speech disturbances, not elsewhere classified | 1·0 |
| Other symptoms and signs involving the  nervous and musculoskeletal systems (R29·6 Tendency to fall) | 3.6 | Streptococcus and staphylococcus as the  cause of diseases classified to other chapters | 1·7 | Vitamin D deficiency | 1·0 |
| Other disorders of urinary system (includes  urinary tract infection and urinary incontinence) | 3.2 | Ulcer of lower limb, not elsewhere classified | 1·6 | Artificial opening status | 1·0 |
| Delirium, not induced by alcohol and other psychoactive substances | 3.2 | Other symptoms and signs involving general  sensations and perceptions | 1·6 | Gangrene, not elsewhere classified | 1·0 |
| Unspecified fall | 3.2 | Duodenal ulcer | 1·6 | Symptoms and signs concerning food and fluid intake | 0·9 |
| Superficial injury of head | 3.2 | Hypotension | 1·6 | Other hearing loss | 0·9 |
| Unspecified haematuria | 3.0 | Unspecified renal failure | 1·6 | Fall on and from stairs and steps | 0·9 |
| Other bacterial agents as the cause of diseases  classified to other chapters (secondary code) | 2.9 | Other septicaemia | 1·6 | Fall on same level from slipping, tripping and stumbling | 0·9 |
| Other symptoms and signs involving cognitive functions and awareness | 2.7 | Personal history of other diseases and conditions | 1·5 | Thyrotoxicosis [hyperthyroidism] | 0·9 |
| Abnormalities of gait and mobility | 2.6 | Respiratory failure, not elsewhere classified | 1·5 | Scoliosis | 0·9 |
| Other cerebrovascular diseases | 2.6 | Exposure to unspecified factor | 1·5 | Dysphagia | 0·8 |
| Convulsions, not elsewhere classified | 2.6 | Other arthrosis | 1·5 | Dependence on enabling machines and devices | 0·8 |
| Somnolence, stupor and coma | 2.5 | Epilepsy | 1·5 | Agent resistant to penicillin and related antibiotics | 0·8 |
| Complications of genitourinary prosthetic  devices, implants and grafts | 2.4 | Osteoporosis without pathological fracture | 1·4 | Osteoporosis with pathological fracture | 0·8 |
| Intracranial injury | 2.4 | Fracture of femur | 1·4 | Other diseases of digestive system | 0·8 |
| Fracture of shoulder and upper arm | 2·3 | Fracture of lumbar spine and pelvis | 1·4 | Cerebral Infarction | 0·8 |
| Other disorders of fluid, electrolyte and acid- base balance | 2·3 | Other disorders of pancreatic internal  secretion | 1·4 | Calculus of kidney and ureter | 0·7 |
| Other joint disorders, not elsewhere classified | 2·3 | Abnormal results of function studies | 1·4 | Mental and behavioural disorders due to use  of alcohol | 0·7 |
| Volume depletion | 2·3 | Chronic renal failure | 1·4 | Other medical procedures as the cause of abnormal reaction of the patient | 0·7 |
| Senility | 2·2 | Retention of urine | 1·3 | Abnormalities of heart beat | 0·7 |
| Care involving use of rehabilitation procedures | 2·1 | Unknown and unspecified causes of morbidity | 1·3 | Unspecified acute lower respiratory infection | 0·7 |
| Unspecified dementia | 2·1 | Other disorders of kidney and ureter, not  elsewhere classified | 1·3 | Problems related to life-management  difficulty | 0·6 |
| Other fall on same level | 2·1 | Unspecified urinary incontinence | 1·2 | Other abnormal findings of blood chemistry | 0·6 |
| Problems related to medical facilities and other health care | 2·0 | Other degenerative diseases of nervous system, not elsewhere classified | 1·2 | Personal history of risk-factors, not elsewhere  classified | 0·5 |
| Vascular dementia | 2·0 | Nosocomial condition | 1·2 | Open wound of forearm | 0·5 |
| Superficial injury of lower leg | 2·0 | Other and unspecified injuries of head | 1·2 | Depressive episode | 0·5 |
| Cellulitis | 2·0 | Symptoms and signs involving emotional  state | 1·2 | Spinal stenosis (secondary code only) | 0·5 |
| Blindness and low vision | 1·9 | Transient cerebral ischaemic attacks and related syndromes | 1·2 | Disorders of mineral metabolism | 0·4 |
| Deficiency of other B group vitamins | 1·9 | Problems related to care-provider dependency | 1·1 | Polyarthrosis | 0·4 |
| Problems related to social environment | 1·8 | Other soft tissue disorders, not elsewhere  classified | 1·1 | Other anaemias | 0·4 |
| Parkinson's disease | 1·8 | Fall involving bed | 1·1 | Other local infections of skin and subcutaneous tissue | 0·4 |
| Syncope and collapse | 1·8 | Open wound of head | 1·1 | Nausea and vomiting | 0·3 |
| Fracture of rib(s), sternum and thoracic spine | 1·8 | Other bacterial intestinal infections | 1·1 | Other noninfective gastroenteritis and colitis | 0·3 |
|  |  |  |  | Fever of unknown origin | 0·1 |

**Charlson Comorbidity Index (CCI)**

The Charlson Comorbidity Index was devised by assessing 607 admissions in one centre during one month. The comorbidities of these patients were recorded on admission and these patients were followed for one year. The comorbidities associated with the highest odds of one year mortality were combined into a weighted score, the CCI. In the original paper, the CCI was stratified into groups with scores of 0, 1-2, 3-4, and ≥5.

| **Condition** | **Points** |
| --- | --- |
| Myocardial infarct | 1 |
| Congestive heart failure | 1 |
| Peripheral vascular disease | 1 |
| Cerebrovascular disease | 1 |
| Dementia | 1 |
| Chronic pulmonary disease | 1 |
| Connective tissue disease | 1 |
| Ulcer disease | 1 |
| Mild liver disease | 1 |
| Diabetes | 1 |
| Hemiplegia | 2 |
| Moderate or severe renal disease | 2 |
| Diabetes with end organ damage | 2 |
| Any tumor | 2 |
| Leukemia | 2 |
| Lymphoma | 2 |
| Moderate or severe liver disease | 3 |
| Metastatic solid tumor | 6 |
| AIDS | 6 |

Johns Hopkins Adjusted Clinical Groups frailty defining illnesses

The Johns Hopkins Adjusted Clinical Groups system is a software application that is used to identify at risk patients on an administrative level. The system uses diagnoses and pharmacy codes from the patient file to identify cost risk, readmission and hospitalisation risk, among others. Within the system is a frailty marker, which is a dichotomous. In the ACG, the presence of 1 or more of the 10 frailty defining illnesses was associated with double the risk of hospitalisation and double the total healthcare costs.

| **Frailty Defining Illnesses** |
| --- |
| Malnutrition and/or Catabolic illness |
| Dementia |
| Severe Vision Impairment |
| Decubitus Ulcer |
| Major Problems of Urine Retention or Control |
| Loss of Weight |
| Absence of Faecal Control |
| Social Support Needs |
| Difficulty in Walking |
| Falls |

**Risk Analysis Index**

The risk analysis index (RAI) was devised to assess risk in elective surgery. The RAI was adapted from the Minimum Data Set (MDS) Mortality Risk Index–Revised (MMRI-R), which is a set of 50 frailty related variables. From these, the 12 variables most associated with 6month mortality were then extracted and formed the RAI (4 questions are used to assess activities of daily living, thus 15 questions in total). This formed the RAI-Clinical (RAI-C).


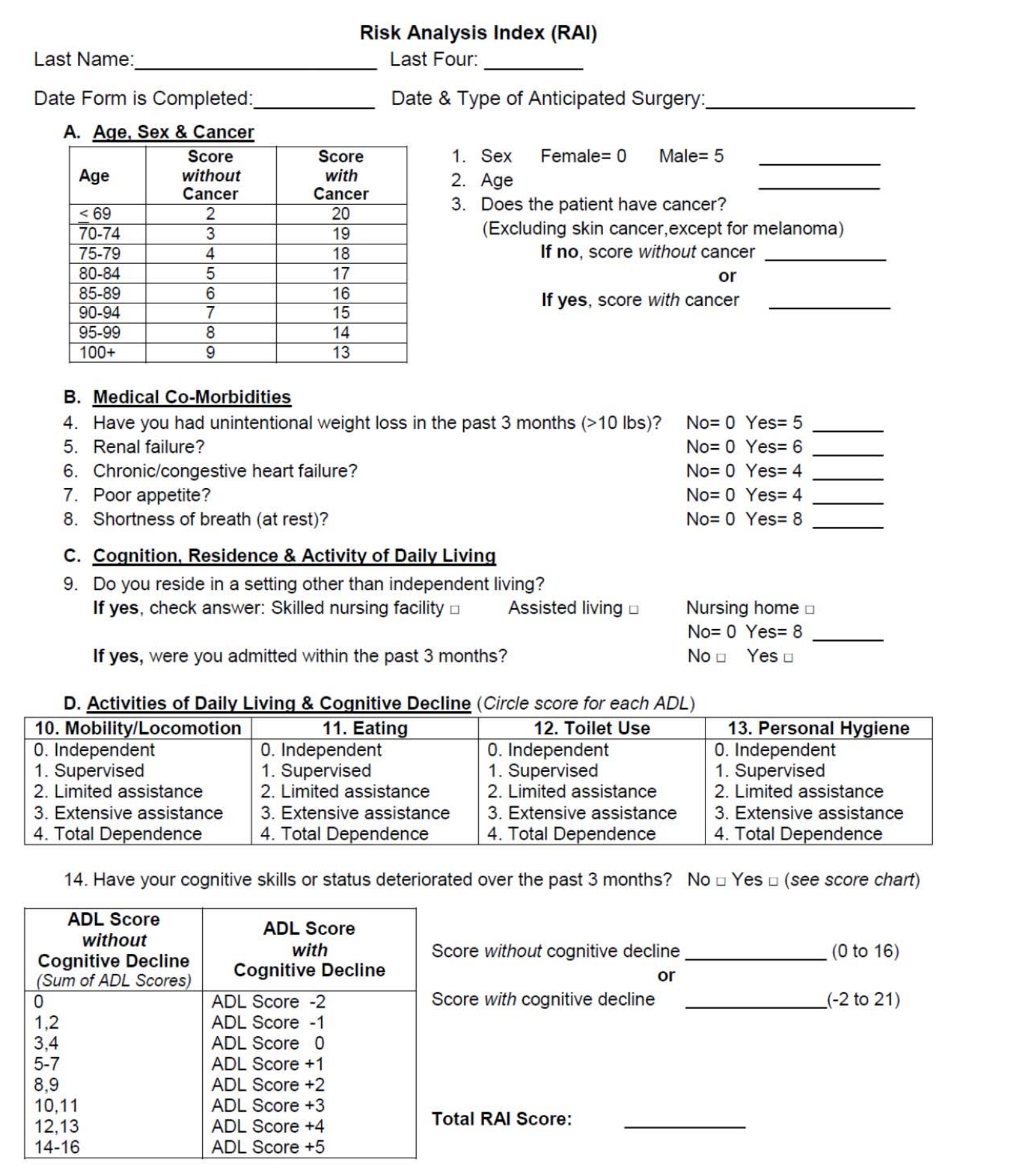


As some of the questions of the RAI-C can not be applied retrospectively with specific databases, specifically the Veterans Affairs or American College of Surgeons National Surgical Quality Improvement Projects (VASQIP/ACS-NSQIP) datasets, an alternative index was created, a modified version of the RAI-C, that could be completed retrospectively with the data available (with some statistical interactions). This was the RAI-Adminstrative (RAI-A).

| **RAI-A Variable** | **Score** |
| --- | --- |
| Male sex | +5 |
| Cancer (except non-melanoma skin cancer) | See Age |
| Age | See RAI-C (scoring depends on cancer) |
| Weight loss | +5 |
| Renal failure | +6 |
| Heart failure | +4 |
| Poor appetite | +4 |
| Shortness of breath at rest | +8 |
| Dependent living | +8 |
| Cognitive deterioration | See Activities of daily living |
| Activities of daily living | Without Cognitive Decline  +16 = totally dependent  +8 = partially dependent  +0 = independent  With Cognitive Decline  +21 = totally dependent  +10 = partially dependent  -2 = independent |

Hall DE, Arya S, Schmid KK, et al. Development and initial validation of the Risk Analysis Index for measuring frailty in surgical populations. JAMA Surg. Published online November 23, 2016. doi:10.1001/jamasurg.2016.4202

**Bibliography**

1. Velanovich V, Antoine H, Swartz A, et al (2013) Accumulating deficits model of frailty and postoperative mortality and morbidity: its application to a national database. J Surg Res 183:104–110. https://doi.org/10.1016/j.jss.2013.01.021

2. Subramaniam S, Aalberg JJ, Soriano RP, Divino CM (2018) New 5-Factor Modified Frailty Index Using American College of Surgeons NSQIP Data. J Am Coll Surg 226:173. https://doi.org/10.1016/j.jamcollsurg.2017.11.005

3. Gilbert T, Neuburger J, Kraindler J, et al (2018) Development and validation of a Hospital Frailty Risk Score focusing on older people in acute care settings using electronic hospital records: an observational study. Lancet Lond Engl 391:1775–1782. https://doi.org/10.1016/S0140-6736(18)30668-8

4. Charlson ME, Pompei P, Ales KL, MacKenzie CR (1987) A new method of classifying prognostic comorbidity in longitudinal studies: development and validation. J Chronic Dis 40:373–383. https://doi.org/10.1016/0021-9681(87)90171-8

5. Johns Hopkins Bloomberg School of Public Health The Johns Hopkins ACG System Version 11.0 Technical Reference Guide. John Hopkins Univeristy, Baltimore, MD

6. Hall DE, Arya S, Schmid KK, et al (2017) Development and Initial Validation of the Risk Analysis Index for Measuring Frailty in Surgical Populations. JAMA Surg 152:175–182. https://doi.org/10.1001/jamasurg.2016.4202
